# *Streptococcus pneumoniae* Invades Erythrocytes and Utilizes Them to Evade Human Innate Immunity

**DOI:** 10.1371/journal.pone.0077282

**Published:** 2013-10-23

**Authors:** Masaya Yamaguchi, Yutaka Terao, Yuka Mori-Yamaguchi, Hisanori Domon, Yuuki Sakaue, Tetsuya Yagi, Kunihiko Nishino, Akihito Yamaguchi, Victor Nizet, Shigetada Kawabata

**Affiliations:** 1 Department of Cell Membrane Biology, Institute of Scientific and Industrial Research, Osaka University, Ibaraki, Osaka, Japan; 2 Department of Oral and Molecular Microbiology, Osaka University Graduate School of Dentistry, Suita, Osaka, Japan; 3 Department of Pediatrics, University of California San Diego, La Jolla, California, United States of America; 4 Division of Microbiology and Infectious Diseases, Niigata University Graduate School of Medical and Dental Sciences, Chuo-ku, Niigata, Japan; 5 Center of National University Hospital for Infection Control, Nagoya University Hospital, Nagoya, Aichi, Japan; Universidad Andres Bello, Chile

## Abstract

*Streptococcus pneumoniae,* a Gram-positive bacterium, is a major cause of invasive infection-related diseases such as pneumonia and sepsis. In blood, erythrocytes are considered to be an important factor for bacterial growth, as they contain abundant nutrients. However, the relationship between *S. pneumoniae* and erythrocytes remains unclear. We analyzed interactions between *S. pneumoniae* and erythrocytes, and found that iron ion present in human erythrocytes supported the growth of *Staphylococcus aureus*, another major Gram-positive sepsis pathogen, while it partially inhibited pneumococcal growth by generating free radicals. *S. pneumoniae* cells incubated with human erythrocytes or blood were subjected to scanning electron and confocal fluorescence microscopic analyses, which showed that the bacterial cells adhered to and invaded human erythrocytes. In addition, *S. pneumoniae* cells were found associated with human erythrocytes in cultures of blood from patients with an invasive pneumococcal infection. Erythrocyte invasion assays indicated that LPXTG motif-containing pneumococcal proteins, erythrocyte lipid rafts, and erythrocyte actin remodeling are all involved in the invasion mechanism. In a neutrophil killing assay, the viability of *S. pneumoniae* co-incubated with erythrocytes was higher than that without erythrocytes. Also, H_2_O_2_ killing of *S. pneumoniae* was nearly completely ineffective in the presence of erythrocytes. These results indicate that even when *S. pneumoniae* organisms are partially killed by iron ion-induced free radicals, they can still invade erythrocytes. Furthermore, in the presence of erythrocytes, *S. pneumoniae* can more effectively evade antibiotics, neutrophil phagocytosis, and H_2_O_2_ killing.

## Introduction

Severe community-acquired pneumonia reported to be the most common cause of death from infection in developed countries [1]. *Streptococcus pneumoniae* is a Gram-positive bacterium and the main cause of community acquired pneumonia worldwide. The pathogen is estimated to be responsible for the deaths of at least 800,000 children each year from pneumococcal disease [2]. In addition, antimicrobial resistance among *S. pneumoniae* strains is increasing throughout the world [3]. *S. pneumoniae* has been categorized into at least 91 serotypes based on the antigenic property of its capsule polysaccharide, while a capsule-conjugated vaccine against a subset of pneumococcal serotypes has shown considerable benefits [4]. However, it has also been reported that serotypes not targeted by the vaccine are increasing [5], [6], while another study showed that *S. pneumoniae* can adapt to clinical interventions over a remarkably short period of time because of a high rate of recombination [7].

*S. pneumoniae* has a variety of virulence factors that contribute to its ability to cause disease, including the secreted toxin pneumolysin (Ply) [8]. Ply is a member of the cholesterol-dependent cytolysin family, a large group of proteins that attack cholesterol-containing membranes, which form ring-shaped pores and become localized in pneumococcal cell walls [9]. A peculiar property of *S. pneumoniae* is its tendency to spontaneously undergo autolysis, with the major autolysin an N-acetyl-muramyl-L-alanine amidase termed LytA [10]. This autolysin degrades peptidoglycan in the pneumococcal cell wall, while it has also been reported that LytA-negative *S. pneumoniae* mutants showed reduced virulence in murine models of pneumonia and bacteraemia [8]. Pneumococcal sortase A is a member of a group of enzymes found in a variety of Gram-positive bacteria that mediate the covalent attachment of proteins containing an LPXTG motif to the cell wall [11]. Through cell wall anchoring of these proteins, sortase A contributes to adhesion to and invasion of human epithelial cells, as well as imparting protection against phagocytic clearance of Gram-positive pathogens including *S. pneumoniae*. Although various strategies for colonization of host epithelial surfaces have been elucidated, the behavior of *S. pneumoniae* in blood remains poorly understood.

Erythrocytes, with a concentration of approximately 5×10^9^ cells/ml, comprise 40–50% of blood volume, and are a key component for the transfer of oxygen and carbon dioxide for cellular respiration. Erythrocytes are also considered to be an important factor in regard to bacterial growth, as they contain abundant nutrients, especially iron, which is required for life in nearly all forms [12]. However, iron interacts with superoxide and hydrogen peroxide (H_2_O_2_) to generate a highly reactive and extremely damaging hydroxyl radical [12]. In addition, a case of transfusion-transmitted *S. pneumoniae* infection caused by contaminated erythrocytes has been reported [13]. However, the relationship between *S. pneumoniae* and erythrocytes has received little attention.

In this study, we provide evidence for the first time that iron in erythrocytes partially inhibits pneumococcal growth using a free-radical-based mechanism, and also that *S. pneumoniae* is able to invade human erythrocytes. Furthermore, we present findings from a neutrophil bactericidal assay showing that the survival rate of *S. pneumoniae* in cultures with erythrocytes was increased by 3-fold as compared to those without erythrocytes. Our results reveal a previously unknown infection strategy employed by *S. pneumoniae*, and also suggest that evasion of the host immune system is facilitated by the pathogen’s use of erythrocyte components and invading human erythrocytes during infection.

## Materials and Methods

### Bacterial Strains and Reagents

*S. pneumoniae* strain D39 (NCTC 7466) was purchased from the National Collection of Type Cultures. *S. pneumoniae* strain R6, which is an unencapsulated derivative D39, was kindly provided by Dr. Shin-ichi Yokota (Sapporo Medical University, Japan). *S. pneumoniae ply*-negative strain R6 and *lytA*-negative mutant strain R6 have been described [14], [15]. Inactivation of the *srtA* gene by double crossover recombination was performed as previously reported [14], [16]. The primers used are shown in Table 1. *S. pneumoniae* and *S. aureus* were grown in Tryptic Soy broth (Becton Dickinson, USA) or 5% sheep blood-Tryptic Soy agar plates with spectinomycin (500 µg/ml) added to the medium for mutant strain selection, while *Escherichia coli* strain XL-10 Gold (Stratagene, USA) was grown in Luria-Bertani broth (Sigma, USA) or on Luria-Bertani agar plates, supplemented with 100 µg/ml of ampicillin or spectinomycin.

**Table 1:** PCR primers used in this study.

Designation	Sequence (5′ to 3′)	Reference
srtAu EcoF	GAATTCTGGATCAGGACGAGTTCACTGC	This study
srtAu BamR	GGATCCCATTATGCTTCACCTTCTGTTTCG	This study
srtAd XbaF	TCTAGATAATACAAATCAGTGAAATCAT	This study
srtAd HindR	AAGCTTCAGTGGCGAAGCATATTTCCAAC	This study
PlyKOu EcoF	GAATTCGTAGCTCTTTATTTGCCTTTTCC	[14]
PlyKOu BamR	GGATCCTCGATAACAACAAACTCATCGG	[14]
PlyKOd XbaF	TCTAGAGGACAATACAGAAGTGAAGGC	[14]
PlyKOd HindR	AAGCTTCTAGTCATTTTCTACCTTA	[14]
lytAs KpnF	GGGGTACCGTCTGGGGTGTTATTGTAGATAG	[15]
lytAs BamR	CGGGATCCCCTGCTTCATCTGCTAGATTGCG	[15]
lytAi XbaF	GCTCTAGAGCCGAAAACGCTTGATACAGGG	[15]
lytAi PstR	AACTGCAGCCGTCTGGTTTGAGGTAGTACCAGCC	[15]

### Isolation of Human Neutrophils and Erythrocytes

Neutrophils were prepared as previously described [14], [17]. Briefly, 10 ml of heparinized blood was obtained from healthy donors and mixed 1∶1 with phosphate buffered saline containing 3% dextran T500. After incubation at room temperature for 60 min, the supernatant was layered on Ficoll-Paque (GE Healthcare, USA). After centrifugation at 450×*g* for 20 min, layers containing erythrocytes and neutrophils were collected. Residual erythrocytes were lysed by hypotonic shock, and then the cells were suspended in RPMI 1640. Cell viability was monitored using the trypan blue exclusion technique and cells were counted with a hemocytometer. Fresh normal erythrocytes were obtained by drawing heparinized blood from volunteer donors, then washed 3 times in RPMI 1640 to remove the buffy coat and used as required.

### Assays of Growth with Erythrocytes

Growth of *S. pneumoniae* strain R6, D39 and *S. aureus* strain Cowan I with erythrocytes and erythrocyte lysates was determined by counting viable CFU. Erythrocytes were suspended to 5.0×10^9^ cells/ml in RPMI 1640. Bacterial cells (∼1.0×10^2^ CFU/well) were added to erythrocytes with or without a final mixture of 1 mM 2,2′-bipyridyl (Sigma), 1 mM 2-ethyl-2-thiopseudourea, hydrobromide (S-Ethyl-ITU, Calbiochem, Germany), 100 µM EUK-8 (Calbiochem), or 150 µM MnTBAP (Calbiochem) for 2, 4, or 6 hours at 37°C in 5% CO_2_. In a growth assay using hemoglobin (Hb), bacterial cells (∼1.0×10^2^ CFU/well) were incubated with or without a final mixture of 10 mg/ml hemoglobin (Sigma) and/or 1 mM 2,2′-bipyridyl in RPMI 1640 for 2, 4, or 6 hours at 37°C in 5% CO_2_. The concentrations of these inhibitors were previously shown to prevent or minimize reactions [18], [19], [20], [21], [22]. The mixtures were serially diluted and plated in TS blood agar. Following incubation, CFU values were determined.

Erythrocyte lysates were prepared using a sonicator. Erythrocytes were suspended to 5.0×10^9^ cells/ml in RPMI1640 and sonicated for 10 minutes on ice. To remove disrupted cell membrane debris, the mixture was centrifuged and the supernatant passed through a 0.20-µm filter. Lysates after filtering were used as membrane-free preparations, while sonicated erythrocytes were used as the membrane-containing counterpart.

### Confocal Fluorescence Microscopic Analysis

Confocal microscopic analysis was performed as previously described [14], [17]. Briefly, fresh erythrocytes (1×10^7^ cells) were infected with *S. pneumoniae* strain R6 or D39 (2.5×10^5^ CFU) for 1 hour, then fixed with 2% glutaraldehyde-RPMI 1640. To observe the localization of *S. pneumoniae,* the bacterial cells were stained with SYTOX Green and erythrocytes were visualized using Alexa Fluor 594 phalloidin (Life Technology, USA). Stained bacteria and erythrocytes were observed using an LSM 510 confocal laser scanning microscope equipped with an α-Plan-fluor 100×/1.45 oil objective lens or a BZ-9000 fluorescent microscopic analyzing system (Keyence, Japan). The obtained images were analyzed with LSM 510 software, version 3.2 SP2 (Carl Zeiss, Germany).

### Scanning Electron Microscopic Analysis

Strains R6 and D39 (2.5×10^6^ CFU) was mixed separately with fresh human blood samples (2 ml) and incubated at 37°C for 30 minutes, then fixed with 2% glutaraldehyde-RPMI 1640 for 1 hour at room temperature and washed with distilled water, after which the samples were dehydrated with 100% *t*-butyl alcohol and freeze-dried. Finally, the samples were coated with platinum and examined using an emission-SEM (JSM-6390LVZ with SEM control user interface software version 8.16; JEOL Ltd., Japan).

### Erythrocyte Invasion Assay

The bactericidal invasion of human erythrocytes was quantified using standard procedures with minor modifications, as previously described [16], [23]. Briefly, erythrocytes were pretreated with or without 5 mM metyl-β-cyclodextrin (MβCD) or 20 µM cytochalasin D for 30 minutes at 4°C, then used for examinations after washing twice. MβCD is able to disrupt lipid rafts by depleting cholesterol [24], [25]. Erythrocytes were added to 96-well plates at a density of 5×10^7^ cells per well and infected with 1×10^4^ CFU of bacteria per well (multiplicity of infection, 1∶5000) for 1 hour. To quantify bacterial invasion, cells incubated with *S. pneumoniae* for 1 hour were washed 3 times and incubated for 1 hour in RPMI 1640 medium containing penicillin G (100 units/ml), then washed again, lysed, and plated to determine the number of invaded *S.* *pneumoniae* organisms.

### Animal Infection

*S. pneumoniae* strain D39 was infected directly into the lungs of mice using a high-pressure syringe Model FMJ-250 with a MicroSprayer Model IA-1C (Penn-Century, Inc., USA) inserted into the trachea. To reduce damage to the lungs by liquid, the organisms (2.5×10^7^ CFU per mouse) were infected as an aerosol using a high-pressure syringe. Three days after infection, mice were euthanized, then the lungs were collected and stained with hematoxylin and eosin (HE) for microscopic examinations. At least 10 microscopic fields (1,000-fold magnification) in the lungs of each mouse (n = 3) were observed and the numbers of bacteria associated with erythrocytes were counted.

### Neutrophil Killing Assay

*S. pneumoniae* strain R6 or D39 was grown to the mid-log phase and resuspended in RPMI1640. Next, 10 µl of bacteria (R6∶2.2×10^2^ CFU, D39∶1.4×10^2^ CFU) was combined with 90 µl of fresh human neutrophils (1.0×10^5^ cells) with or without erythrocytes (5.0×10^7^ cells), and/or 10% human blood serum or heat-inactivated human blood serum, then the mixtures were incubated while being rotated at 37°C for 1, 2, or 3 hours. Viable cell counts were determined by plating lysed and diluted samples onto blood agar.

### H_2_O_2_ Killing Assay

*S. pneumoniae* strain R6 or D39 was grown to the mid-log phase and resuspended in RPMI 1640. Next, 1.0×10^2^ CFU/10 µl of bacteria was incubated with or without erythrocytes (5.0×10^6^ or 5.0×10^8^ cells/90 µl) for 30 minutes, then H_2_O_2_ was added (final concentration 0%, 0.03%; 8.82 mM, or 0.30%; 88.2 mM). Viable cell counts were determined by plating diluted samples onto blood agar following 30 minutes of incubation.

### Ethics Statement

All mice experiments were conducted in accordance with animal protocols approved by the Animal Care and Use Committees at Osaka University Graduate School of Dentistry. Blood was obtained via venopuncture from healthy Japanese volunteers under written informed consent according to a protocol approved by the institutional review boards of Osaka University Graduate School of Dentistry, Niigata University Graduate School of Medical and Dental Sciences, and the University of California San Diego. Blood cultures from the patients were obtained after they had given written informed consent according to a protocol approved by the Nagoya University Hospital Ethics Review Board.

## Results

### Iron Inhibits Growth of *S. pneumoniae* with Human Erythrocytes

**Figure 1:**
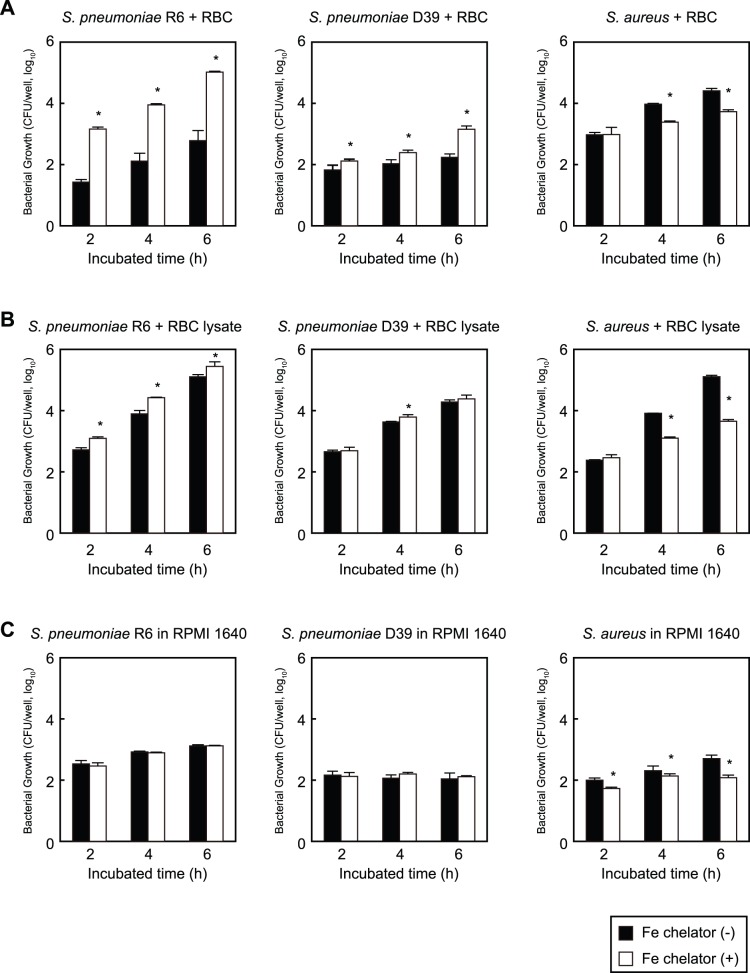
Effects of erythrocytes and iron ions on *S. pneumoniae* growth. *A*. Growth of *S. pneumoniae* strains R6 and D39, and *S. aureus* strain Cowan-I in the presence of human erythrocytes with or without an iron chelator. Bacterial cells were incubated for 2, 4, and 6 hours at 37°C in a 5% CO_2_ atmosphere. *B*. Growth of *S. pneumoniae* strains R6 and D39, and *S. aureus* strain Cowan-I in erythrocyte intracellular solution (erythrocyte lysates without membrane) with or without an iron chelator for 2, 4, and 6 hours at 37°C in a 5% CO_2_ atmosphere. *C*. Growth of *S. pneumoniae* strains R6 and D39, and *S. aureus* strain Cowan-I in RPMI 1640 medium with or without an iron chelator for 2, 4, and 6 hours at 37°C in a 5% CO_2_ atmosphere. The experiments were performed 3 times and data shown represent the mean of 3 wells from a representative experiment. S.D. values are represented by vertical lines.

We speculated that erythrocytes contribute to the proliferation of *S. pneumoniae* and examined their role in pneumococcal growth. Approximately 50 colony forming units (CFU) of *S. pneumoniae* strain R6, D39, or *S. aureus* strain Cowan I were prepared with erythrocytes, then the mixtures were cultured with or without an iron chelator, 2,2′-bipyridyl, at 37°C. Next, 1 mM 2,2′-Bipyridyl was used to deplete or limit the amounts of iron ion [18]. Bacterial growth was determined by counting the number of viable CFU on blood agar. We found that addition of the iron chelator inhibited the growth of *S. aureus* with erythrocytes (Fig. 1) and chelator-treated *S. aureus* did not grow on Tryptic Soy agar (data not shown). This finding is consistent with previous reports, which noted that iron is essential for *S. aureus* [26] and its growth in the presence of erythrocytes was significantly inhibited by an iron chelator [18]. In contrast, we found that the growth of *S. pneumoniae* was significantly enhanced by 54-, 69-, and 173-fold (R6), or 2.0-, 2.3-, and 8.3- fold (D39) when cultured in the presence of the iron chelator for 2, 4, and 6 hours, respectively (Fig. 1*A*). Next, we cultured both bacterial strains with erythrocyte lysates. The growth of *S. aureus* grown with erythrocyte lysates was similar to that when incubated with intact erythrocyte cells, while a lower level of growth inhibition of *S. pneumoniae* was seen when grown with erythrocyte lysates as compared to intact erythrocyte cells (Fig. 1*B*). The bacterial growth activity of *S. pneumoniae* with erythrocyte lysates and the iron chelator was increased by 2.4-, 3.4-, and 2.2-fold (R6), or 1.1-, 1.4-, and 1.3- fold (D39) after 2, 4, and 6 hours, respectively, as compared to without the chelator. In contrast, addition of the iron chelator did not have a significant effect on the growth of *S. pneumoniae* strains R6 and D39 when cultured in RPMI 1640 medium without erythrocytes (Fig. 1*C*). These results indicate that *S. pneumoniae* does not require a rich iron ion environment in comparison to *S. aureus*, and instead that iron ions inhibit *S. pneumoniae* growth.

### Erythrocytes Inhibit Growth of *S. pneumoniae* via Iron-induced Free Radical-based Mechanism

**Figure 2:**
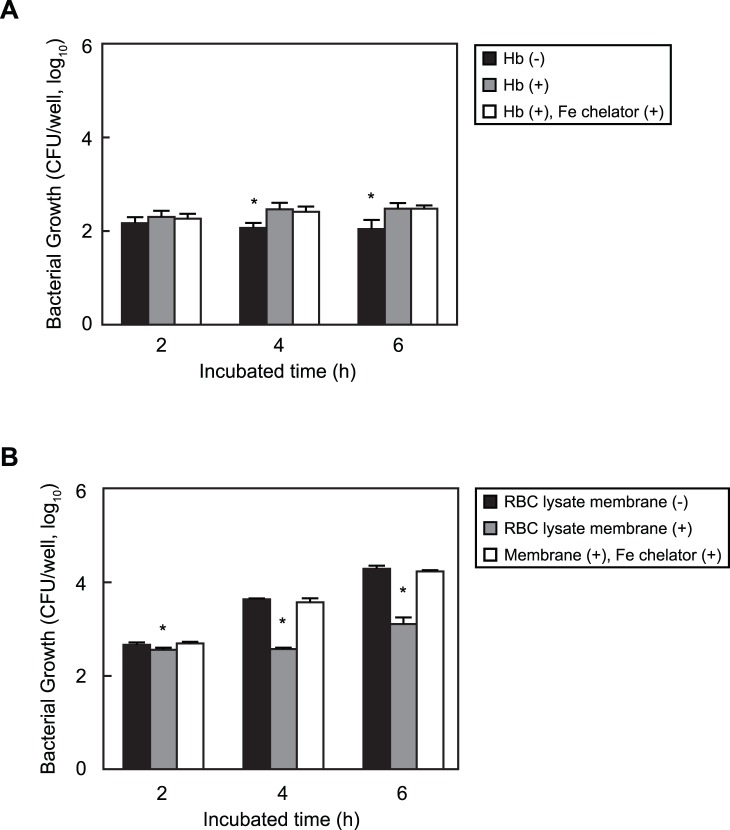
Erythrocyte intracellular solution inhibits pneumococcal growth in presence of erythrocytes with the membrane. *A.* Growth of *S. pneumoniae* strain D39 in RPMI 1640 medium with or without human hemoglobin and/or an iron chelator for 2, 4, and 6 hours at 37°C in a 5% CO_2_ atmosphere. *B.* Growth of *S. pneumoniae* strain D39 in erythrocyte lysates with or without the erythrocyte membrane and/or an iron chelator for 2, 4, and 6 hours at 37°C in a 5% CO_2_ atmosphere. The experiments were performed 3 times and data shown represent the mean of 3 wells from a representative experiment. S.D. values are represented by vertical lines.

We next investigated whether erythrocyte iron ions inhibit pneumococcal growth. In the presence of Hb, pneumococcal growth was not inhibited but rather increased as compared to without Hb (Fig. 2*A*). Next, we compared pneumococcal growth in the presence of erythrocyte lysates containing or lacking the erythrocyte membrane. When cultured with lysates with the membrane, growth was significantly inhibited in comparison to that observed in lysates without the membrane. In addition, the iron chelator diminished the degree of growth inhibition by lysates containing the erythrocyte membrane (Fig. 2*B*). These results indicate that pneumococcal growth inhibition requires both iron ions and the erythrocyte membrane.

**Figure 3:**
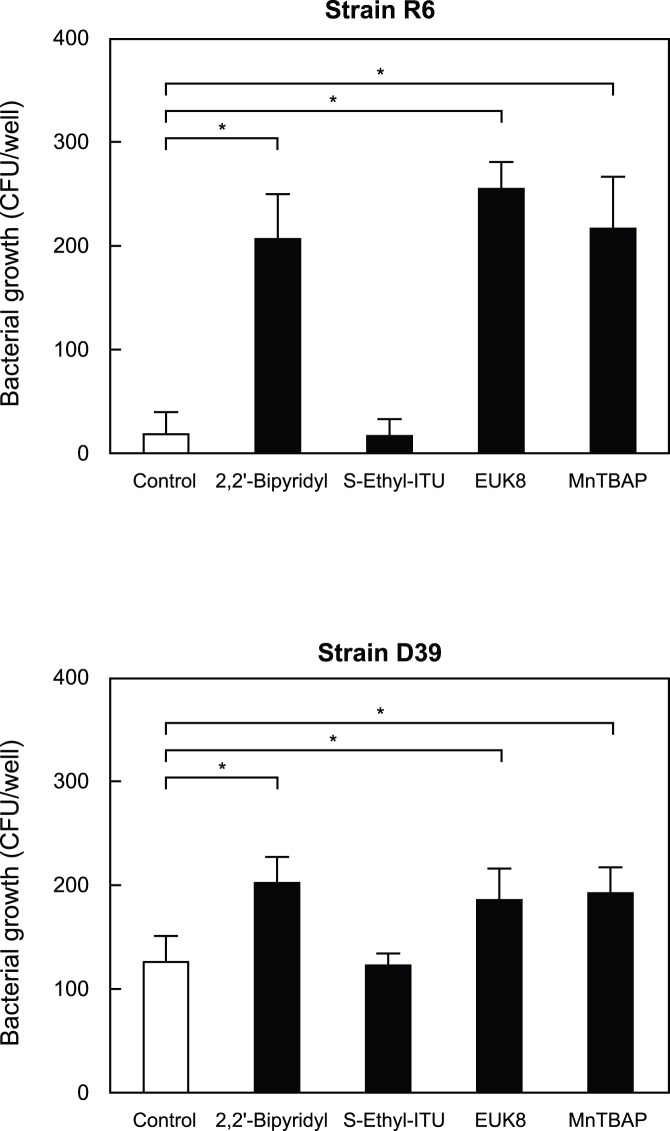
Erythrocytes inhibit pneumococcal growth by reactive oxygen species-related mechanism. *S. pneumoniae* cells (∼1×10^2^ CFU, 10 µl) were added to erythrocytes (5×10^9^ cells/ml, 190 µl) with or without 1 mM 2,2′-bipyridyl (iron chelator), 1 mM S-ethyl-ITU (nitric oxide synthase inhibitor), 100 µM EUK8 (synthetic catalytic free radical scavenger), or 150 µM MnTBAP (superoxide dismutase mimetic) for 2 hours at 37°C in a 5% CO_2_ atmosphere. Next, each mixture was serially diluted and plated on TS blood agar. Following incubation, CFU values were determined. *Significant difference (*P*<0.005) between mean values, as determined with a Mann-Whitney *U*-test. The experiments were performed 3 times and data are shown as the mean of 6 wells from a representative experiment. S.D. values are represented by vertical lines.

Although iron is essential for the growth of a large proportion of bacteria, iron ions mediate the generation of free radicals [12], [27], while superoxide anion, hydroxyl radical, and nitric oxide are generated in human erythrocyte free radical metabolism pathways [28]. A number of different electron transport processes are present in the erythrocyte membrane, and previous reports have shown that some of these electron transport chains function as electron donors and can generate superoxide anion [28], [29]. We speculated that free radicals would inhibit the growth of *S.* *pneumoniae* when grown with erythrocytes and examined the mechanism of pneumococcal growth inhibition using oxidative stress inhibitors. *S. pneumoniae* was incubated with erythrocytes in the presence of an iron chelator (2,2′-bipyridyl), nitric oxide synthase inhibitor (S-Ethyl-ITU), free radical scavenger (EUK-8), or superoxide dismutase mimetic (MnTBAP) for 2 hours. S-Ethyl-ITU is an inhibitor of all isoforms of nitric oxide synthases [19], while EUK-8 has high superoxide dismutase- and catalase-mimic activities, and oxyradical scavenging activities [20], and MnTBAP functions as a superoxide dismutase mimetic but does not scavenge nitric oxide [21], [22]. Each mixture was plated on tryptic soy blood agar, and the increase in number of *S. pneumoniae* CFU recovered was determined. The growth rates of *S. pneumoniae* when incubated with the iron chelator, free radical scavenger, and superoxide dismutase mimetic were at least 11-fold (R6) or 1.5-fold (D39) greater than that of *S. pneumoniae* grown with S-Ethyl-ITU or without inhibitors (Fig. 3). Based on these results, we concluded that erythrocytes inhibit pneumococcal growth via a free radical-based mechanism.

### *S. pneumoniae* Invades Human Erythrocytes

**Figure 4:**
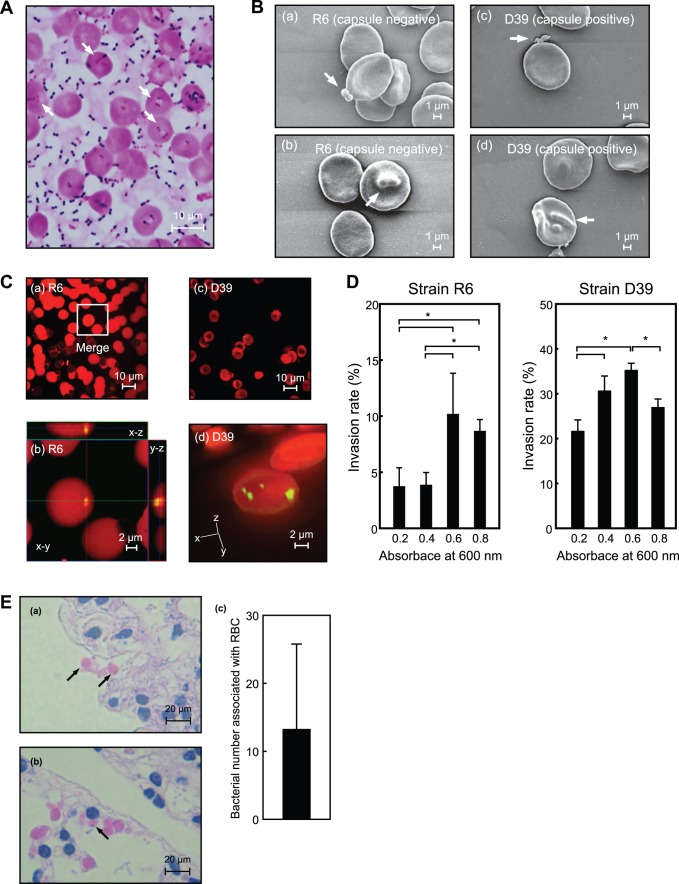
*S. pneumoniae* invasion of human erythrocytes. *A*. Gram staining of cultures of blood obtained from a patient with invasive pneumococcal pneumonia. A blood sample was obtained from a splenectomized patient with pneumococcal bacteremia and meningitis. We observed that some of the *S. pneumoniae* cells in the sample adhered to or invaded erythrocytes. *B*. SEM analysis of *S. pneumoniae* in blood. *S. pneumoniae* cells (arrows) were incubated in human whole blood for 30 minutes at 37°C. Strains R6 and D39 adhered to (a, c) and invaded (b, d) erythrocytes in human blood. *C*. Confocal fluorescence microscopic analysis of *S. pneumoniae* strains R6 (a, b) and D39 (c, d) incubated with human erythrocytes for 30 minutes at 37°C. (a, c) Erythrocytes were visualized using Alexa Fluor 594 Phalloidin. *S. pneumoniae* organisms were stained using SYTOX green. (b) Boxed areas from panel (a), along with x–z and y-z projections. (d) 3D analysis of image from panel (c) showing erythrocytes invaded by *S. pneumoniae*. *D*. Rate of *S. pneumoniae* invasion of erythrocytes. The numbers of invaded bacteria were determined as described in the Experimental Procedures section. *Significant difference (*P*<0.005) between mean values, as determined with a Mann-Whitney *U*-test. The experiments were performed 3 times and data are shown as the mean of 6 wells from a representative experiment. S.D. values are represented by vertical lines. *E*. Histopathological examinations of infected mice lung tissues. Tissues were excised from sites of infection after 72 hours, then fixed, embedded in paraffin, and stained with hematoxylin-eosin solution. (a) and (b) were obtained from individual mice. Arrows indicate association of *S.* *pneumoniae* with erythrocytes. (c) Numbers of bacteria associated with erythrocytes per field. Data shown represent the mean of 10 fields from a representative mouse. S.D. values are represented by vertical lines.

Gram staining of blood cultures infected with invasive *S.* *pneumoniae* bacteria under clinical laboratory test conditions showed that some of the pneumococci became associated with human erythrocytes (Fig. 4*A*). In order to further investigate the invasion of erythrocytes by *S. pneumoniae*, mixtures of the bacteria and erythrocytes were analyzed in detail using a scanning electron microscope (SEM) and confocal fluorescence microscopy. SEM analysis was performed with human blood samples incubated for 1 hour with unencapsulated *S. pneumoniae* strain R6 or its encapsulated parent strain D39 (Fig. 4*B*), which revealed that the organisms adhered to (Fig. 4*B* *a*, *c*) and invaded (Fig. 4*B* *b*, *d*) erythrocytes in human blood, regardless of the capsule phenotype. In fluorescence analysis with confocal microscopy performed with human erythrocytes incubated for 1 hour with *S. pneumoniae* strain R6 or D39 Z-stack analysis clearly showed that both strains invaded erythrocytes (Fig. 4*C*).

Next, we performed pneumococcal invasion assays to determine the percentages of intracellular bacteria in the early to late growth phases of *S. pneumoniae*. We added *S. pneumoniae* strain R6 or D39 (∼1.0×10^4^ CFU) in each phase of growth to erythrocytes, and found that the invasion rates of R6 were 3.8%, 3.9%, 10.2%, and 8.7%, while those of D39 were 21.6%, 30.6%, 35.2%, and 26.9% at an absorbance (600 nm) of 0.2, 0.4, 0.6, and 0.8, respectively (Fig. 4*D*). These results indicated a significant difference for the invasion rate of *S. pneumoniae* between the early and late growth phases. In addition, *S. pneumoniae* grown with erythrocytes evaded antibiotic killing by invading those erythrocytes.

We also observed the interaction between *S. pneumoniae* and erythrocytes *in vivo* in lungs obtained from infected mice. For these studies, we used encapsulated strain D39, since non-encapsulated strain R6 is rapidly eliminated by the immune system of the mice. *S. pneumoniae* were injected in an intratracheal manner into C57BL/6 mice, then the lungs were obtained after euthanasia and stained with HE. Microscopic examinations revealed that *S.* *pneumoniae* organisms were associated with or had invaded erythrocytes in the lungs (Fig. 4*E*).

### *S. pneumoniae* Invade RBC via Actin-remodeling and Lipid Rafts

**Figure 5:**
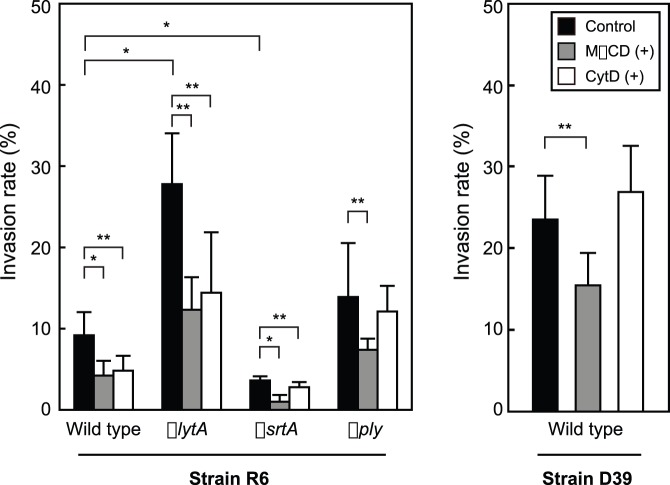
Involvement of lipid rafts and actin remodeling in erythrocyte invasion by *S. pneumoniae*. Erythrocytes were pretreated with or without 5βCD or 20 µM cytochalasin D for 30 minutes at 4°C, then *S. pneumoniae* cells were added and incubated for 1 hour at 37°C in a 5% CO_2_ atmosphere. The numbers of invaded bacteria were determined as described in the Experimental Procedures section. The experiments were performed 3 times and data shown represent the mean of 6 wells from a representative experiment. S.D. values are represented by vertical lines. *P<0.005; **P<0.05.

Gram-positive bacteria invade host epithelial and endothelial cells via actin-remodeling caused by interactions between bacterial cell wall anchoring proteins and host receptors [30]. The cholesterol-dependent cytolysin of *S. pneumoniae*, termed Ply, was previously shown to cause cholesterol-dependent actin remodeling in SH-SY5Y human neuroblastoma cells [31]. In addition, lipid rafts, a cholesterol- and sphingolipid-enriched micro-domain in cell membranes, play crucial roles in the invasion of host cells by various pathogens [32]. To determine the invasion mechanisms, we performed invasion assays using several inhibitors and *S. pneumoniae* gene-deficient strains. We prepared *S. pneumoniae* strains at an absorbance (600 nm) of 0.6–0.7. The *S. pneumoniae ΔlytA* strain showed an invasion rate 3.0-fold greater as compared to the wild-type strain, while the *ΔsrtA* strain showed a 61% reduction in invasion efficiency. Furthermore, encapsulated *S. pneumoniae* strain D39 showed an invasion frequency 2.5-fold greater compared to its unencapsulated derivative strain R6. On the other hand, no significant difference between the *Δply* and the wild-type strains (Fig. 5). Lipid raft disruption of erythrocytes using the cholesterol-extracting agent MβCD resulted in a 64% (R6) and 34% (D39) reduction in invasion efficiency for the wild-type pneumococcal strains. In addition, inhibition of erythrocyte actin polymerization by cytochalasin D resulted in 2-fold drop in invasion efficiency for the wild-type R6 strain. In contrast, cytochalasin D did not inhibit invasion by strain D39. As for the *ΔsrtA* strain, lipid raft disruption and inhibition of actin polymerization resulted in invasion efficiencies of 28% and 77% of control, respectively. These results indicated that an LPXTG motif containing proteins, lipid rafts, and actin remodeling are all involved in the erythrocyte invasion pathway of *S. pneumoniae*.

### *S. pneumoniae* Evades Neutrophil Killing in the Presence of Erythrocytes

**Figure 6:**
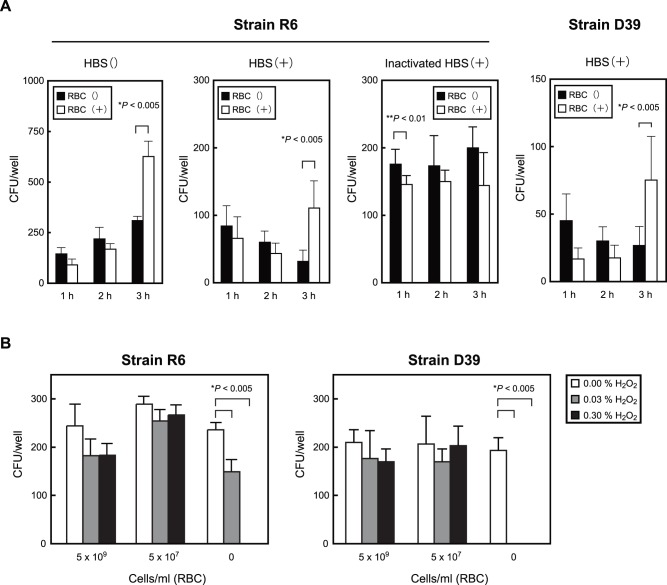
Erythrocytes inhibit killing of *S. pneumoniae*. *A*. Inhibition of killing by neutrophils. *S. pneumoniae* cells (R6∶2.2×10^2^ CFU/well, D39∶1.4×10^2^ CFU/well) were incubated with human neutrophils (1×10^5^ cells/well), then erythrocytes (5.0×10^7^ cells/well) and/or 10% human blood serum (HBS) or heat-inactivated HBS were added to the mixture. Viable CFU were counted following 1, 2, and 3 hours of incubation. *B.* Inhibition of killing by H_2_O_2_. *S. pneumoniae* cells were incubated in 0%, 0.03%, or 0.30% H_2_O_2_-RPMI 1640, then viable CFU were counted following 1, 2, and 3 hours of incubation. The experiments were performed 3 times and data shown represent the mean of 6 wells from a representative experiment. S.D. values are represented by vertical lines.

We performed bactericidal assays to determine whether erythrocytes have effects on neutrophil bacterial killing. *S. pneumoniae* cells and neutrophils were incubated with or without erythrocytes in fresh or heat-inactivated human serum, then antiphagocytic activities were determined based on the viability of the *S. pneumoniae* organisms. In the absence of serum, the viability of *S. pneumoniae* with erythrocytes was higher than that without erythrocytes after 3 hours. In contrast, in the presence of fresh serum, recovery of viable *S. pneumoniae* strain R6 cells grown without erythrocytes declined gradually over a 1 to 3 hour time frame., In contrast, when the assay was performed in the presence of erythrocytes CFU recovery of pneumococci was significantly higher at the late time point. Parallel assays performed with strain D39 revealed a similar pattern. In the presence of heat-inactivated serum, the viability of *S. pneumoniae* strain R6 was not significantly changed (Fig. 6*A*). These results indicate that *S. pneumoniae* evades neutrophil opsonophagocytosis in the presence of erythrocytes.

Generation of H_2_O_2_ is one of the antimicrobial mechanisms of neutrophils. In addition, *S. pneumoniae* dies by exposure to its own production of H_2_O_2_ in a stationary phase [33]. For these reasons, we assessed the effect of erythrocytes on *S. pneumoniae* susceptibility to H_2_O_2_. We exposed *S. pneumoniae* strains R6 and D39 to a range of H_2_O_2_ concentrations in RPMI 1640, and found that 0.30% and 0.03% H_2_O_2_ killed 100% and 37% (R6), and 100% and 100% (D39), respectively, of those bacteria, whereas H_2_O_2_ killing was nearly completely lost in the presence of erythrocytes (Fig. 5*B*). These results indicate that H_2_O_2_ killing is inhibited not only by erythrocyte invasion, but also by erythrocyte catalase activity against H_2_O_2_.

## Discussion

Iron is essential for the life of virtually all organisms [12]. However, the present results indicate that *S. pneumoniae* can grow under an iron-depleted condition, which, in contrast, inhibits the growth of *S. aureus*. Ong *et al*. investigated the growth of *S. pneumoniae* strain D39 in chemically defined medium without iron and that without manganese, and found no significant difference [34]. It is reasonable that *S. pneumoniae* has a low requirement for iron, because this organism lacks a respiratory chain and possesses only a few enzymes that contain iron-sulfur clusters [34], [35], [36], [37]. Furthermore, in this study, we showed that iron from intact erythrocytes partially inhibited the growth of *S. pneumoniae*, even though this bacterium is one of the most common Gram-positive pathogens isolated from patients with bloodstream infections [38]. In relation to their physiologic role, human erythrocytes contain abundant oxygen, a potential source of free radicals. Furthermore, erythrocytes are rich in iron ions, which can induce hydroxyl radicals from H_2_O_2_ and/or superoxide anion via Fenton and Harber-Weiss reactions [28]. Although hydroxyl radicals have an extremely short half-life (1×10^−9^ seconds at 37°C), they are highly toxic and cause damage to virtually all types of macromolecules, including DNA, proteins, carbohydrates, and lipids [29], [39]. Enzymatic antioxidants such as superoxide dismutase and catalase do not directly eliminate hydroxyl radicals, whereas they are able to eliminate superoxide anion and H_2_O_2_, which are sources of hydroxyl radicals. Thus, hydroxyl radicals may be the main factor involved in growth inhibition of *S. pneumoniae* in the presence of erythrocytes.

Pathogens have been reported to use two different strategies to avoid contact with neutrophils [40]. First, many pathogenic bacteria and fungi are able to inhibit recruitment of neutrophils to the site of infection. For example, lack of neutrophil migration to the site of infection has been frequently noted in severe *Streptococcus pyogenes* infection cases, as streptococcal proteases degrade interleukin-8, and complements C5a and C3b [40], [41]. Second, these pathogenic organisms reside in regions inaccessible to phagocytes. However, it has been observed that many neutrophils are recruited to areas around *S. pneumoniae* organisms. On the other hand, *S. pneumoniae* are able to escape from innate immunity and can spread deep into tissues in cases with severe pneumococcal infections. Thus, we speculated that *S. pneumoniae* has a function to equip itself with molecules or mechanisms to evade neutrophilic immunity. In the present study, we found that *S. pneumoniae* evades antibiotics, neutrophils, and H_2_O_2_ killing in the presence of human erythrocytes. It is generally accepted that invasion of erythrocytes provides bacterial pathogens with a number of advantages, including protection from the immune system, reduction in efficacy of antibiotics treatment, and nutritional benefits. Thus, erythrocytes are considered to provide shelter for *S. pneumoniae* in spite of the disadvantage that iron ions partially reduce bacterial growth. Furthermore, it is possible that this invasion ability is related to the difference between the minimum inhibitory concentration *in vitro* and that in host blood, because penicillin G does not kill *S. pneumoniae* organisms after they have invaded erythrocytes.

*S. pneumoniae* organisms grown in culture broth spontaneously die when reaching the stationary phase, which is a phenomenon dependent on the *spxB* gene and its by-product H_2_O_2_ [33]. Interestingly, H_2_O_2_ production by *S. pneumoniae* provides an advantage in competition with other species *in vivo* [42]. In the present study, H_2_O_2_ killing of *S. pneumoniae* was inhibited in the presence of erythrocytes, suggesting that the pathogen eliminates H_2_O_2_ using catalase present in erythrocytes, which may explain why *S. pneumoniae* organisms lacking catalase show a competitive advantage by producing H_2_O_2_ and carrying the *spxB* gene.

*S. pneumoniae* adheres to erythrocytes in a complement- and antibody-dependent process called immune adherence, which enhances its phagocytosis by neutrophils [43], [44]. Immune adherence is mediated by the complements C3b, C4b, and C1q, as well as mannose-binding lectin on immune complexes that interact with the CR1 receptor on the surface of erythrocytes [45]. In the present study, *S. pneumoniae* adhered to and invaded erythrocytes in the absence of human serum, while the pathogen evaded neutrophil killing in the presence of human serum and erythrocytes, indicating that erythrocyte invasion by *S. pneumoniae* occurs independent of immune adherence.

In our experiments, we found no significant difference in erythrocyte-invasion between wild-type and *Δply* strains of *S. pneumoniae*. Ply attaches to the cell membrane in a cholesterol-dependent manner and then oligomerization to form pores on the membrane [46]. At a glance, it seems unusual that bacteria producing cytolysin can also invade erythrocytes. However, *S. pneumoniae* does not destroy host cells through Ply activity when the multiplicity of infection is low. In fact, the pathogen has been reported to invade the human lung epithelial cell line A549 and evade antibiotics killing, while Ply has been shown to cause LDH release in cells [16], [47], [48]. Another interesting result is that the *ΔlytA* strain showed a 3-fold greater level of invasion as compared to the wild-type strain. One possibility is that the *ΔlytA* strain does not degrade its own cell wall, resulting in a greater number of LPXTG motif-containing proteins remaining on the cell surface to more effectively function as invasion factors as compared to the wild-type strain. Another simple possibility is that LytA and/or degraded peptidoglycan directly inhibit the interaction. Although it has been reported that some *Mycoplasma* species invade host erythrocytes in animals, the invasion mechanism or pathway remains unclear [49], [50]. In the present study, we found that *S. pneumoniae* invaded human erythrocytes using a variety of factors, including lipid rafts, actin remodeling, and LPXTG motif-containing proteins. Therefore, the mechanism of erythrocyte invasion shares some similarities with that of epithelial cell invasion [51], [52].

The present findings also demonstrated that human erythrocytes partially inhibit pneumococcal growth by generating iron-induced free radicals. However, *S. pneumoniae* was found able to invade human erythrocytes and evade innate immunity. Interestingly, though transfusion-transmitted pneumococcal infection caused by erythrocytes has been reported, cultures of blood and swabs from the antecubital fossae, nose, and throat of affected patients were found to be negative for pneumococcal antigens, with urinary testing providing the same results [13]. Thus, it is possible that erythrocytes function as a Trojan horse for the pathogen following pneumococcal invasion. The present novel findings have the potential to cause a paradigm shift in the understanding of sepsis, transfusion transmitted infections, and clinical blood test results, as well as the choice of antibiotics in affected patients.
