# Correction: *Streptococcus pneumoniae* Invades Erythrocytes and Utilizes Them to Evade Human Innate Immunity

**DOI:** 10.1371/annotation/de14e562-d899-48da-b677-ca61cca2cf5b

**Published:** 2014-01-15

**Authors:** Masaya Yamaguchi, Yutaka Terao, Yuka Mori-Yamaguchi, Hisanori Domon, Yuuki Sakaue, Tetsuya Yagi, Kunihiko Nishino, Akihito Yamaguchi, Victor Nizet, Shigetada Kawabata

Errors were introduced during the preparation of this article for publication. The first sentence of figure legend 5 is incorrect. The correct first sentence of figure legend 5 is: Erythrocytes were pretreated with or without 5 µM MβCD or 20 µM cytochalasin D for 30 minutes at 4°C, then "S". 

In Figure 5, 'delta' and 'beta' were replaced with white boxes. The correct version of Figure 5 can be viewed here: http://plosone.org/corrections/pone.0077282.g005.cn.tif

In Figure 6, '-' signs have been omitted. The correct version of Figure 6 can be viewed here: http://plosone.org/corrections/pone.0077282.g006.cn.tif

